# Evidence of psychological and biological effects of structured Mindfulness‐Based Interventions for cancer patients and survivors: A meta‐review

**DOI:** 10.1002/pon.5771

**Published:** 2021-07-28

**Authors:** Juliana Pedro, Sara Monteiro‐Reis, Carina Carvalho‐Maia, Rui Henrique, Carmen Jerónimo, Eunice R. Silva

**Affiliations:** ^1^ Psychology Service Portuguese Oncology Institute of Porto (IPOP) Porto Portugal; ^2^ Cancer Biology & Epigenetics Group Research Center Portuguese Oncology Institute of Porto (CI‐IPOP) Porto Portugal; ^3^ Department of Pathology Biobank Portuguese Oncology Institute of Porto (IPOP) Porto Portugal; ^4^ Department of Pathology and Molecular Immunology Institute of Biomedical Sciences Abel Salazar University of Porto (ICBAS‐UP) Porto Portugal

**Keywords:** anxiety, cancer, immune response, inflammatory‐related markers, MBCT, MBSR, meta‐review, mindfulness, oncology, psychological

## Abstract

**Objective:**

A large number of studies have been conducted exploring the effects of mindfulness programs on health outcomes, such as psychological and biological outcomes. However, there is substantial heterogeneity among studies and, consequently, in the systematic reviews/meta‐analyses. Since clinical practice is massively informed by evidence on review studies, our main objective was to summarize the reported evidence regarding the effects of structured mindfulness‐based programs on psychological, biological, and quality‐of‐life outcomes in cancer patients.

**Methods:**

We conducted a meta‐review, using a literature search from inception to June 2020 in several electronic databases using a combination of keywords including MBSR, MBCT, cancer, and meta‐analysis OR “systematic review” (PROSPERO registration CRD42020186511).

**Results:**

Ten studies met the eligibility criteria and were included. The main findings were beneficial small to medium effect sizes of Mindfulness‐Based Stress Reduction (MBSR)/Mindfulness‐Based Cognitive Therapy (MBCT)/Mindfulness‐Based Cancer Recovery (MBCR) on psychological health, such as anxiety, depression, stress, and quality of life. A beneficial effect was found for biological outcomes, albeit based on a reduced number of studies. Studies were moderate homogenous regarding the intervention, population, and outcomes explored. Results on long‐term follow‐up seem to indicate that the effects tend not to be maintained, namely in shorter follow‐ups (6 months).

**Conclusions:**

This meta‐review brings a broad perspective on the actual evidence regarding MBSR/MBCT/MBCR. We expect to contribute to future project design, focused on developing high‐quality studies and exploring the moderating effects that might contribute to biased results, as well as exploring who might benefit more from MBSR/MBCT/MBCT interventions.

## 1 INTRODUCTION

Research on the effects of mindfulness on health has proliferated, with the number of publications increasing exponentially since the late 1990s.^1^ However, a large number of articles do not guarantee that the evidence gathered on the effectiveness of mindfulness is strong since research on mindfulness is complex, with multiple methodological issues that can compromise the quality of evidence.^2^

One of the most relevant problems concerns the definition of mindfulness. Successive adaptations to better adjust to theories and psychological intervention programs and simplifications resulting from its practice in contexts not guided by science resulted in different versions of the concept. A universal technical definition of mindfulness and its underlying aspects has not yet been found.^2^, ^3^ Another problem results from the diversity of ways to carry out interventions based on mindfulness. These can range, for example, from simple guided meditation to intervention programs that include a mindfulness component, programs with different components or timelines, adaptations to standardized programs, or differences in methods of teaching and practicing mindfulness, which makes it difficult to compare them in clinical studies. The term Mindfulness‐Based Interventions (MBIs) has emerged to include interventions with mindfulness components in conjunction with other interventions or theoretical approaches. Some interventions include some components of mindfulness, but the basis of the therapy is not mindfulness, such as Dialectical Behaviour Therapy (DBT) and Acceptance and Commitment Therapy (ACT),^4^, ^5^ these so‐called Mindfulness‐Informed Interventions.^6^ Until a consensus is reached on the concept of mindfulness, research on its effectiveness would benefit from comparing standardized mindfulness intervention protocols, which could be replicated, and the resulting evidence would only apply to these programs.

Mindfulness was introduced in Medicine and Psychology by Jon Kabat Zinn in 1979 as a standardized program to reduce stress in the Stress Reduction Clinic at The University of Massachusetts. This Mindfulness‐Based Stress Reduction (MBSR) program integrates a classic view of mindfulness accordingly to its Buddhist roots. Kabat‐Zinn defined mindfulness as “the awareness that emerges through paying attention on purpose, in the present moment, and nonjudgmentally to the unfolding of experience moment by moment” (p. 145).^7^

MBSR program is based on intensive training in mindfulness meditation and mindful hatha yoga and preconizes present‐centered non‐judgmental acceptance, awareness, and attentiveness compassion.^8^ MBSR is based on four foundations: awareness of the body, feeling tone, mental states, and mental contents and is based in formal and informal practices: “mindful movement (gentle hatha yoga with an emphasis on mindful awareness of the body); the body scan (designed to systematically, region by region, cultivate awareness of the body—the first foundation of mindfulness—without the tensing and relaxing of muscle groups associated with progressive relaxation); and sitting meditation (awareness of the breath and systematic widening the field of awareness to include all four foundations of mindfulness: awareness of the body, feeling tone, mental states, and mental contents)” (p. 188).^4^

The gold‐standard model which is in the base of other two main programs broadly used in clinical settings, namely the Mindfulness‐Based Cognitive Therapy (MBCT) and the Mindfulness‐Based Cancer Recovery (MBCR), is the MBSR 8‐week course, involving 20–26 h of formal meditation in group classes of 1.5–2.5 h each, one all‐day (6 h) class, and home practice (about 45 min/day, 6 days/week).^2^

MBIs have been increasingly used to improve psychological health in diverse psychological and health conditions, for example, inflammatory bowel disease.^9^ In particular, MBSR and MBCT suggest being effective for improving mental health outcomes in people with vascular disease^10^ and with chronic fatigue syndrome.^11^

In the context of cancer, MBIs such as MBSR, MBCT, and MBCR or their modified versions are the most used standardized protocols and have been applied to reduce distress in patients across all stages of the disease.^12^ MBCT was initially created to be a relapse‐prevention therapy for major depression disorder.^13^ It modified the classic structure of MBSR to include components of cognitive–behavioral therapy. The MBCR is an adaptation of the MBSR program to make it more suitable for cancer patients.^14^

Evidence supports that MBIs can improve the psychological health of individuals with cancer,^15^ cancer‐related fatigue,^16^ and, more recently, cancer‐related biomarkers.^17^

Research on the communication between psychological and biological aspects of cancer has been growing over the last few years. There is a growing interest in understanding the interconnections between biological and psychological systems, aiming to find effective ways to improve health outcomes (biological and psychological) in cancer patients and survivors.^17^, ^18^, ^19^

Several studies and, consequently, reviews have summarized the evidence on some of these outcomes. A descriptive review on mindfulness and biomarkers in cancer patients found that participation in MBIs impacts psychological measures and biological parameters (immune function, hypothalamic–pituitary–adrenal axis regulation, and autonomic nervous system activity).^18^ However, this review was not systematic, which imposes a methodological limitation. Sanada and colleagues reviewed the effects of MBIs on biomarkers in cancer and healthy groups and found some evidence of changes in cytokine levels.^17^ The majority of reviews focused exclusively on physiological outcomes or only on psychological outcomes. It brought a challenge to interpret and inform health professional's decision‐making, namely due to the heterogeneity of participants included and incongruence in MBI definition, which might have contributed to different results.

The goal of this meta‐review is to sum all evidence presented in previous reviews investigating the effect of structured MBIs on psychological outcomes (such as anxiety, depression, and stress), quality of life (QoL), as well as biological outcomes (e.g., inflammatory response), focusing on patients with cancer and cancer survivors. Using systematic reviews as a unit of analysis allows addressing broader research questions than those examined in individual systematic reviews, and understanding the diversity present in the existing systematic review literature.^20^ We hope that this work might help clarify the available evidence on the effectiveness of structured mindfulness interventions, identify specific gaps in scientific literature, guide future research, and inform health providers' decision‐making.

## 2 METHODOLOGY

### 2.1 Study design

A meta‐review of reviews was conducted following the PRISMA‐P guidelines (Preferred Reporting Items for Systematic Review and Meta‐Analysis Protocols)^21^ and guidance from Cochrane^20^ and Smith and colleagues^22^ on methodology in conducting a systematic review of systematic reviews of healthcare interventions. The protocol of this meta‐review was pre‐registered in PROSPERO (CRD42020186511).

### 2.2 Search strategy

A systematic search was conducted from inception to June 2020 in the following electronic databases: Cochrane Library; Medline complete, APA PsycArticles, APA PsycInfo, Academic Search Ultimate, Fonte Acadêmica, Psychology and Behavioral Sciences Collection through EBSCO; and Science Citation Index Expanded, Social Sciences Citation Index, Emerging Sources Citation Index through Web of Science. Searches were conducted using broad search terms, keywords, Medical Subject Headings (MESH), and filters for “systematic reviews,” “cancer,” language, and time frame were used and adaptable, if available in the databases. The search expression was a combination of three key themes (using several search terms)^1^: effectiveness of manualized structured mindfulness‐based interventions^2^; population: cancer patients and cancer survivors^3^; systematic review/meta‐analysis methodology.

The preliminary search expression used for Medline was the following: (“Meditation intervention” OR mindfulness OR “Mindful*psychotherap*” OR meditation OR MBIs OR “Mindfulness‐Based Stress Reduction” OR MBSR OR “Mindfulness‐Based Cognitive Therapy” OR MBCT) AND (cancer OR “cancer survivor” OR “cancer recovery” OR “cancer survivorship”) AND (meta‐analysis OR “systematic review” OR review).

### 2.3 Inclusion and exclusion criteria

Systematic reviews addressing the effectiveness of MBIs conducted with adult cancer patients and/or survivors were included. Following Smith^22^ and Cochrane guidelines,^20^ the inclusion criteria were defined so that the included systematic reviews were sufficiently homogenous regarding interventions, population, comparators, and outcome measures. The criteria for inclusion were as follows^1^: being a systematic review or meta‐analysis of randomized controlled trials (RCTs)/effectiveness studies (at least with a control group; even if not randomized)^2^; studies examining the pre‐post effect of a Manualized Structured Mindfulness‐Based Intervention that followed predetermined curricula (e.g., MBCT, MBSR, MBCR) as the main goal; studies that presented diverse interventions types other than mindfulness were only included if they addressed the mindfulness studies in a considerable number in the review synthesis (more than 70% of studies included) and presented the results and conclusions separated for these structured MBIs^3^; addressing the effects on adults with cancer (cancer patients and/or survivors)^4^; reporting at least, one of the following quantitative health outcome measures: psychological outcomes (such as anxiety, depression and stress, fear of cancer recurrence [FCR]); QoL and biologic outcomes (e.g., inflammatory response)^5^; studies written in English, Spanish, French, or Portuguese.

The exclusion criteria were as follows^1^: reviews that addressed other types of mindfulness or meditation‐based techniques (e.g., complementary therapies, yoga solely, tai‐chi; mind‐body therapies focused only on exercise and meditation/yoga)^2^; reviews considered of low‐quality^3^; grey literature: thesis, letters, editorials, posters, and not peer‐reviewed papers^4^; reviews presenting a significant overlap of studies included (see Table S1).

### 2.4 Study screening and selection process

All entries were imported to *Covidence software* (www.covidence.org) This software facilitates the management of databases‐selected entries, search for duplicates, and selection process conducted by independent reviewers. In the first phase, entries were inspected for duplicates. Then, titles and abstracts were screened for eligibility independently by two reviewers (JP and ES). Subsequently, a full‐text evaluation was conducted on those who met the previous criteria. Disagreements at each stage of screening and selection were solved by discussion between the two authors.

### 2.5 Data extraction

One author (JP) conducted the data extraction, which was cross‐checked by the last author (ES). The data extracted included the author, year, country of publication, type of review, database searched, restrictions (e.g., language, dates), interventions addressed, number of studies included, quality assessment of studies included in the review, population/sample characteristics, interventions, primary outcome, and findings. The overlap of systematic reviews based on exactly the same primary studies was resolved by selecting only one of them, being this the most recent, complete, and extensive systematic review. According to the method suggested by Pollock,^20^ since the main goal is not to re‐systematically review the primary studies, only studies where conclusions can be extracted (based on evidence, preferably meta‐analysis results) will be included.

### 2.6 Assessment of review quality

AMSTAR tool^23^, ^24^ was used to assess the quality of the systematic reviews included. This tool was found to be reliable^24^ and has been used in several meta‐reviews.^25^, ^26^ Previous studies using AMSTAR considered scores indicating low quality (score 0–4), moderate quality (score 5–8), and high quality (9–11).^25^, ^26^ According to this criteria, only reviews with a moderate or higher score (5 or above) were included in this meta‐review. The first author evaluated all the studies, and 50% of these were evaluated by the second author.

### 2.7 Strategy for data analysis and synthesis

The information extracted from the included studies was summarized in tables, describing the characteristics of studies included and a summary of the main findings and conclusions. A narrative synthesis approach was used to conduct this meta‐review.^27^ Included studies were categorized, analyzed, and presented according to the outcomes reported (psychological, biological). In addition, results on subgroup analysis and moderators will be described, if available. Data on heterogeneity, sensitivity analysis, and other risk bias will be extracted and summarized for each study included.

## 3 RESULTS

### 3.1 Study screening and selection

**FIGURE 1:**
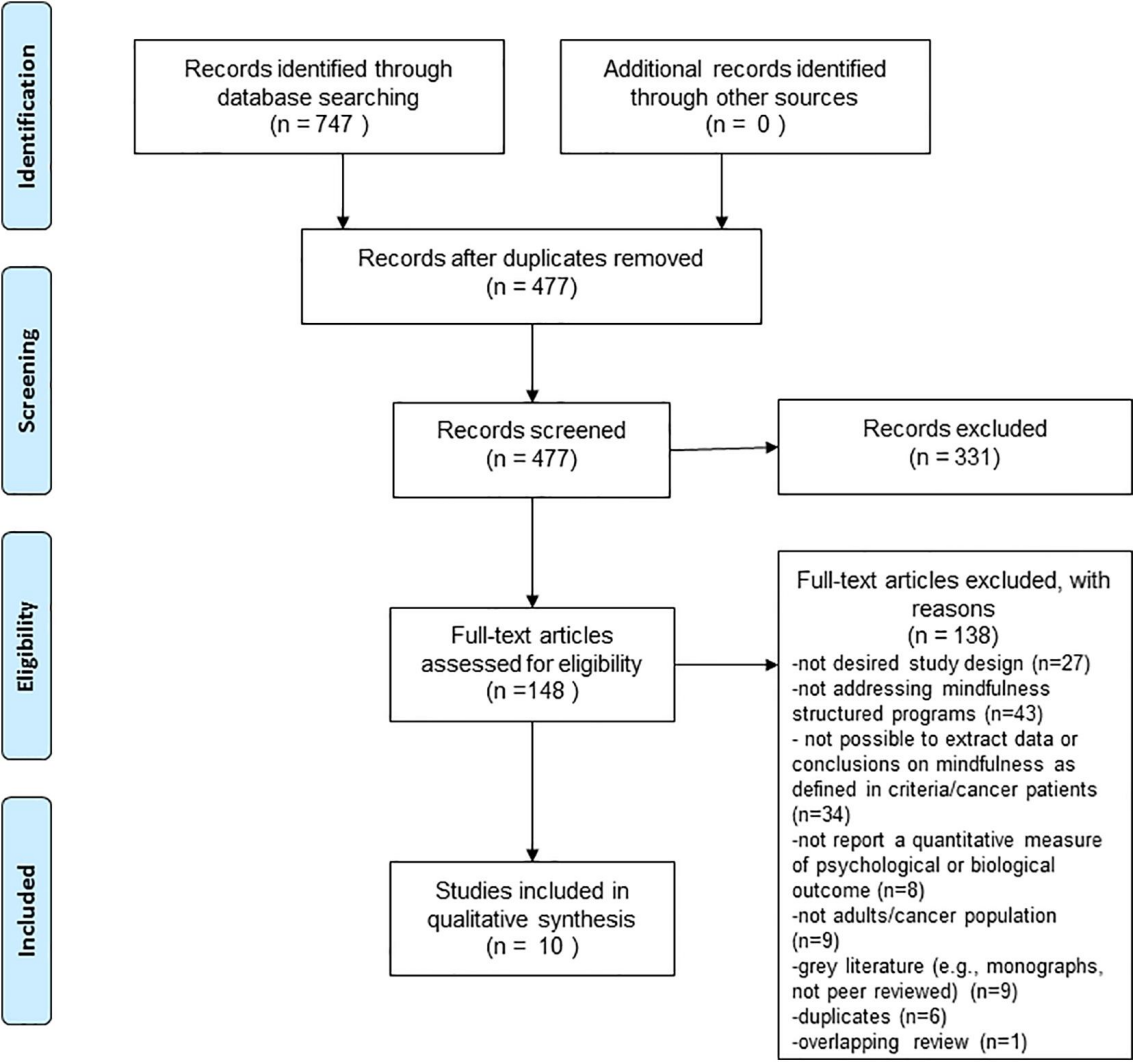
Study flow chart (Moher et al.^28^)

Figure 1 presents the study flow diagram of the search and selection process. The initial search identified 747 entries. After removing duplicates (*n* = 270), 477 studies were screened based on title and abstract. In this phase, 331 were irrelevant. During the full‐text phase, 148 studies were assessed for eligibility. One hundred and thirty‐eight did not meet the inclusion criteria (see Figure 1 for details), and thus, 10 studies were included in this meta‐review.

### 3.2 Overlap between studies

Overlap between studies is summarized in Table S1. One review^29^ included primary studies that were all present in the other reviews, so it was excluded from the present study. The majority of other reviews included presented significant overlap; however, all of them included studies that were only present once; in this sense, no other review was excluded. On average, each study was included in two reviews. The number of reviews that included the same primary study range from one to seven; only four original studies were presented in more than four reviews.

### 3.3 Studies quality

Using AMSTAR tool, studies had a mean quality value of 8.6 (range 5–10), meaning that all included studies were of high quality (all studies were classified 5 or above). The rate of agreement between the first and the second authors was high (all studies were rated as 5 or above by both authors, being the maximum difference between quality rates 3 points).

### 3.4 Characteristics of studies included

Table 1 shows the characteristics of the 10 included studies. Studies were published between 2012 and 2020. The studies were from China (*n* = 2), Germany (*n* = 2), Denmark (*n* = 2), United States (*n* = 1), Singapore (*n* = 1), and Spain (*n* = 1). All studies included used more than two databases for their systematic searches, with Medline, Pubmed, PsycInfo, Cumulative Index to Nursing, and Allied Health Literature (CINAHL), and Cochrane Central Register of Controlled Trials being the most common. Most of the studies were focused on cancer patients/survivors with several cancer types (*n* = 4), with the majority addressing specifically breast cancer patients and survivors (*n* = 6). All studies addressed MBIs as the main goal, namely MBSR/MBCT/MBCR. From the 10 studies included, 2 explored both the effect of mindfulness on psychological and biological outcomes.^30^, ^31^ The most common outcomes were anxiety, depression, and stress. The inclusion criteria for participants' age ranged from 18 to 75, with most studies reporting a mean age around 55 years old. The majority of studies included participants with any cancer stage and any treatment status or adjuvant treatment, with only one excluding metastatic cancer. The follow‐up times ranged between 2 and 24 months, being the most common 6‐month follow‐up.

**TABLE 1:** Characteristics of included studies (*n* = 10)

Author (year, country)	Design	Databases searched	Goal (inclusion and exclusion criteria	Search period	Sample characteristics	Interventions addressed	Control group	Outcome measure (primary)	No. studies included in the original	No. studies meeting criteria	Range follow‐up times
Calero (2018, Spain)	SR	PubMed, CINAHL, and PsycINFO	RCTs with follow‐up	January 2011 to October 2017	1839 women survivors of BC stage 0–III (including those in hormonal therapy)	MBCT/MBSR/MBCR	UC	Psychological	10	9	3 months to 6–12 months after
			Exclusion criteria: chemotherapy/radiotherapy		Age range: 18–75; mean = 55 years						
Cillessen (2019, Denmak)	SR & M	PubMed, Web of Science, PsycINFO, and CINAHL	RCTs	Inception to 10 October 2018	3274 cancer patients and survivors (any cancer location, all stages)	MBCT/MBSR/MBCR	WL	Stress, anxiety, depression	29 RCTs (38 papers)	23	Mean follow‐up: 6 months
					Age range: 46–71; mean = 55 years						
Cramer (2012, Germany)	SR & M	MEDLINE, Cochrane Library, Embase, CAMbase, and PsycINFO	RCTs and BC patients	Inception to November 2011	327 BC patients (including both in chemotherapy/radiation)	MBSR/MBCT	UC, AC, no treatment	QoL, psychological health	3	3	12–24 months
			Exclusion criteria: metastatic cancer		Age range: 50–57.5 years						
Ford (2020, United States)	M	PsycINFO, MEDLINE	RCTs	Inception to December 2017	369 men with cancer (prostate, mixed, lung)	MBSR/MBCT	UC, WL, AC	Psychosocial outcomes	17	9	2–12 months
			Men with cancer of any treatment status (including pretreatment, current treatment, and posttreatment)		369 men (in the nine studies using MBSR, MBCT)						
			At least 10% of the study population was male								
Haller (2017, Germany)	SR & M	PubMed (including MEDLINE), Scopus (including Embase), and Cochrane CENTRAL	RCTs, cluster RCTs, randomized cross over	Inception to October 2016	1709 BC participants (stage 0–IV); the majority not metastatic	MBCT/MBSR	UC, AC	QoL (primary), fatigue, stress, anxiety, depression	14 (10 samples)	10	3–12 months
			MBCT/MBSR (ACT excluded, mindfulness body exercise)		Mean age = 54.3 years old						
					Includes both participants who had completed cancer treatments and who were undergoing adjuvant treatment						
Piet (2012, Denmark)	SR & M	Embase, PubMed, PsycINFO, Web of Science, Scopus, and the Cochrane CENTRAL		Inception to 5 March 2012	1403 cancer patients and survivors, BC (77%), prostate cancer, mixed cancer	MBSR/MBCT	UC, WL	Anxiety, depression, mindfulness skills	22	9 RCTs on meta‐analysis (*n* = 959 participants)	1–12 months; mean post‐test follow‐up: 6.6 months
					Mean age = 55 years old						
Schell (2019, Germany)	SR	Cochrane CENTRAL, MEDLINE, Embase, WHO ICTRP, and ClinicalTrials.gov	RCTs	January 2005–2017	1957 women with BC	Only MBSR	UC	QoL	10	10	6 months to 2 years
			All stages of BC, including metastatic cancer		Age range: 56–59 years old						
					Includes both participants who had completed cancer treatments and who were undergoing adjuvant treatment						
Xunlin (2020, Singapore)	SR & M	PubMed, Embase, Cochrane Library, Scopus, CINAHL, PyscINFO, MEDLINE, ScienceDirect, and Clinical Trials Registry	RCTs	2008–2018	3476 cancer patients and survivors	MBSR/MBCT/MBCR	UC, WL, AC	Psychological and QoL	29	24 on meta‐analysis addressing MBSR/MBCT/MBCR	Not reported
			Cancer patients or survivors aged ≥18 years old		Age range: 38.8–70.7 years old						
			Any type and stage of cancer, and treatment status		BC, prostate, colorectal, lung cancer, mixed cancer						
Zhang (June 2016, China)	SR & M	PubMed, Cochrane Library, EBSCO, Chinese Biomedical Literature Database, and Chinese Digital Journals Full‐text	RCTs	Inception to January 2015	951 BC patients; cancer stage 0–II	MBSR	UC, WL	Physical health, psychological health, and QoL	7	7	Not reported
			Diagnosis of BC regardless treatment status								
Zhang, Qiuxiang (2019, China)	SR & M	Cochrane Library, Cochrane CENTRAL, PsycINFO, Web of science, MEDLINE, Embase, CNKI, and CBM	Trails on MBSR versus control group	February to May 2018	1505 BC patients; a majority of non‐metastatic cancer	MBSR	UC, AC, no treatment	Symptom, QoL	14	14	4 weeks to 24 months
					Mean age = 48.17 years old (range 40.67–56.90)						

Abbreviations: AC, active control group; BC, breast cancer; M, meta‐analysis; MBCR, Mindfulness‐Based Cancer Recovery; MBCT, Mindfulness‐Based Cognitive Therapy; MBSR, Mindfulness‐Based Stress Reduction; QoL, quality of life; RCT, randomized controlled trial; SR, systematic review; UC, usual care; WL, waist‐list control group.

### 3.5 Psychological outcomes related to mental health

In general, most systematic reviews and meta‐analyses concluded that MBSR/MBCT/MBCR had a significant and beneficial effect on anxiety symptoms, depression, stress, and general psychological outcomes.

Calero and colleagues concluded that all studies found a significant improvement in psychological symptoms, including anxiety, depression, stress, and QoL and fear of recurrence.^30^ Cillensen and colleagues^12^ found a small and statistically significant effect of MBSR/MBCT on combined measures of psychological distress at post‐intervention (Hedges' *g* = 0.32; 95% CI [0.22, 0.41]; *P* < 0.001), namely in anxiety [0.22, 0.51; *P* < 0.001], depression [0.18, 0.58], psychological stress [0.22, 0.41], and fear of recurrence [ 0.12, 0.45; *P* < 0.001]; no effect was found for QoL [−0.02, 0.55; *P* = 0.066]. Cramer et al.^31^ also found a small but significant effect in depression (SMD = −0.37; 95%CI [–0.65, −0.08]; *P* = 0.01); significant and moderate effect on anxiety (SMD = −0.51; 95% CI [–0.80, 0.21]; *P* = 0.0009). Significant and beneficial effects of MBSR/MBCT but inconsistent across studies were found in distress and QoL^31^ (data not meta‐analysed). Also, Ford and colleagues^32^ found a small effect (Hedges' *g* = 0.17; 95% CI [0.00, 0.35]; *P* = 0.05) of MBIs compared with meditative movement for all pooled outcomes (anxiety, depression, stress, QoL); however, effects for QOL fell short of statistical significance (Hedges' *g* = 0.16; 95% CI [–0.10, 0.56]; *P* = 0.17).^32^

Compared with usual care, Haller and colleagues^33^ found small but significant post intervention effects of MBSR/MBCT/MBCR on QoL (SMD = 0.21; 95% CI [0.04, −0.39]), stress (SMD = −0.33; 95% CI [–0.61, −0.05]), anxiety (SMD = −0.28; 95% CI [–0.39, −0.16]), and depression (SMD = −0.34; 95% CI [–0.46, –0.21]).

Piet and colleagues^34^ reported meta‐analysis for RCTs and non‐RCTs (without control group) studies separately. Based on our inclusion criteria, only results on RCTs are reported, and the results revealed a significant and moderate effect of MBSR/MBCT/MBCR both for anxiety (Hedges' *g* = 0.37; 95% CI [0.24, 0.50]; *P* < 0.001) and depression (Hedges' *g* = 0.44; 95% CI [0.24, 0.64]; *P* < 0.001).^34^

Another review from Schell et al.^35^ which included 10 RCTs found that MBSR slightly reduces short‐term anxiety (SMD = −0.29; 95% [CI −0.50, −0.08] and depression (SMD = −0.54, 95% [CI −0.86, −0.22]). Xunlin and colleagues^36^ performed a systematic review and meta‐analysis including 29 studies, from those 24 addressed MNSR/MBCT/MBCR. The meta‐analysis on these 24 studies found beneficial effects of MBSR/MBCR on anxiety and depression compared with other interventions (SMD = −0.30, *P* = 0.0003), but intervention effect for MBCT was found to be of shorter statistical significance (SMD = 0 .17, *P* = 0.44), Zhang Jun and colleagues^15^ also found positive effects of MBSR/MBCT in reducing anxiety (SMD = −0.31; 95% CI [−0.46, −0.16]; *P* < 0.0001), depression (SMD = −1.13; 95% CI [−1.85,−0.41]; *P* = 0.002), fear of recurrence (SMD = −0.71; 95% [CI−1.05, −0.38]; *P* < 0.0001), and emotional well‐being (SMD = 0.39; 95% CI [0.19, 0.58]; *P* = 0.0001). Another systematic review and meta‐analysis conducted by Zhang Qiuxiang and colleagues^37^ found beneficial and significant effects of MBSR on wellbeing (SMD = 1.01; 95% CI [0.35, 1.67]; *P* = 0.003), anxiety (SMD = −0.54; 95% CI [−1.01, −0.07]; *P* = 0.02), depression (SMD = −0.61; 95% CI [−1.11, −0.11]; *P* = 0.02), stress (SMD = −0.48; 95% CI [−0.81, −0.15]; *P* = 0.004), and distress (SMD = −0.56; 95% CI [−0.85, −0.26]; *P* = 0.0002). No significant effects of MBSR on QoL were found.^37^

### 3.6 Biological outcomes

The effects of mindfulness on immune‐related biomarkers have also been reported in two studies included in this review.^30^, ^31^

In this context, MBSR/MBCT/MBCR were shown to improve T‐ and natural killer (NK)‐cells activity, as well as an immune recovery (measured by the T helper (Th)‐1/Th‐2 and CD4+/CD8+ ratios) (data described, not pooled effects available).^31^ In another study, increased telomere activity was associated with the implementation of structured MBI.^30^

### 3.7 Results on long‐term effects

Some of the included studies, namely meta‐analysis, conducted subgroup analysis exploring the long‐term effects of MBSR/MBCT/MBCR. The majority found maintained effects at follow‐up times for anxiety and depression. Nonetheless, these results seem to be more consistent and frequent in shorter follow‐ups (6 months comparing with 12 months after intervention).

Calero and colleagues concluded that most studies found a significant maintenance of the beneficial effects of MBSR/MBCT/MBCR 12 weeks later.^30^ Consistent with Cillensen’ study, which found a small and statistically significant pooled effects of MBIs on combined measures of psychological distress were also found at follow‐up (*g* = 0.19; 95% CI = [0.07, 0.30]; *P* < 0.002).^12^ Haller and colleagues^33^ report that effects were maintained up to 6 months after baseline regarding anxiety (SMD = −0.28; 95% CI = [−0.47, −0.09]) and depression (SMD = −0.26; 95% CI = [−0.47, −0.04]); and 12 months after regarding anxiety (SMD = −0.21; 95% CI = [−0.40, −0.03]). Piet et al.^34^ also found maintained effects of MBSR/MBCT at follow up for anxiety (Hedges' *g* = 0.26; 95% CI = [0.10, 0.42]; *P* = 0.002) and depression (Hedges' *g* = 0.19; 95% CI = [0.03, 0.36]; *P* = 0.02), indicating no evidence of heterogeneity between studies but effect sizes were considered less robust in this follow‐up results. Up to 6 months after baseline, Schell and colleagues^35^ found that MBSR had a beneficial effect on slightly reducing anxiety (SMD = −0.28; 95% CI = [−0.49, −0.07]) and depression (SMD = −0.32; 95% CI [−0.58 to −0.06]). Results 12 months after intervention indicating little to no evidence of benefits in anxiety (SMD = −0.09; 95% CI = [−0.35, 0.16.]) and depression (SMD = −0.17; 95% CI = [−0.40, 0.05]).^35^

### 3.8 Moderators

The studies included performed subgroup and other analyses to explore the role of moderators on the effectiveness of MBIS. Cillensen reported that larger effects on psychological distress were found in younger patients when comparing with passive control conditions, shorter follow‐up times.^12^ Cramer found superior effects were found for studies comparing MBSR/MBCT/MBCR with usual care (vs. other active controls) in decreasing depression (SMD: −0.37; 95% CI = [–0.65 to −0.08]; *P* = 0.01; *I* ^2^ = 0%) and anxiety (SMD: −0.51; 95% CI = [–0.80, −0.21]; *P* = 0.0009; *I* ^2^ = 0%).^31^ Haller also analyzed the effects of MBSR/MBCT regarding other active interventions, and results revealed that only significant and small effects post‐intervention for anxiety (SMD = −0.45; 95% CI = [−0.71, −0.18]) and depression (SMD = −0.39; 95% CI = [−0.65, −0.14]).^33^ MBSR/MBCT/MBCR were found to be equally effective both for patients under adjuvant treatment for cancer and survivors.

### 3.9 Publication bias, heterogeneity, and quality of the evidence

The majority of studies assessed the methodological quality of studies included, assessed heterogeneity and other risks of bias relevant. In sum, the majority reported some concerns regarding quality and heterogeneity between studies was frequent. Some of the studies could not report the risk of bias and conduct sensitivity analysis due to a low number of studies included.

Cramer et al. assessed heterogeneity and concluded that age was similar among studies but found some heterogeneity regarding treatment phase (some patients were in adjuvant treatments, others finished the treatment)^31^; sensitivity analysis and publication bias were not possible to assessed due to the small number of studies included in the meta‐analysis. Cillensen reported a slightly small publication bias.^12^ Haller et al.^33^ used the Cochrane risk of bias tool and reported that findings were robust to methodological bias since results did not change when only studies with low risk of bias were considered; however, the number of studies was low, and the reported effects were below the criteria to be considered clinically relevant. Piet and colleagues reported a low‐to‐moderate heterogeneity between included studies, and no evidence of publication bias was found for short‐term results; RCTs reported successful randomization (no baseline differences on characteristics); however, the results on follow‐up were considered less robust.^34^ Schell et al. noted that all studies included were at high risk of performance, detection, and selection bias. Even though, results on short‐term and medium‐term effects for anxiety were considered high‐certainly evidence and for depression moderate‐certainly evidence, using GRADE tool.^35^ Xunlin et al.^36^ also reported substantial heterogeneity of studies. However, publication bias and sensitivity analysis did not reveal significant changes in results. These analyses, and subgroup analysis (nor reported here), were based on the total number of studies, which included other MBIS, not MBSR/MBCT/MBCR, and for this reason, are not referred to in the present study. Sensitivity analysis performed by Zhang and colleagues^15^ found that results were unchanged and statistical differences remain between the control group and intervention group for depression (SMD = −0.42; 95% CI [−0.57, −0.27]; *P* = 0.00). The authors reported that the methodological quality of the included studies was low to moderate and subgroup analysis was not possible due to the low number of studies.^15^ Zhang and colleagues also assessed the risk of bias with the Newcastle and obtained a score of 6 (range 5–7), showing good quality generally.^37^

## 4 DISCUSSION

To the best of our knowledge, this is the first meta‐review summarizing the results of this amount of evidence, focused on examining the high‐quality evidence about the effectiveness of MBSR/MBCT/MBCR on cancer patients and survivors. This meta‐review only retained high‐quality review studies, which had included studies using a control group. Furthermore, only studies with the main goal of summarizing the effects of MBSR/MBCT/MBCR were included only after the study's quality assessment and overlapping analysis.

The result of this meta‐review supports the effectiveness of MBSR/MBCT/MBCR in psychological outcomes. We found a consistent and significant effect of MBSR/MBCT/MBCR on decreasing anxiety, depression, and stress. Although the effect size reported were small to medium, these results are in line with most of the literature on the effectiveness of mindfulness, both for healthy individuals,^38^ patients with a chronic illness other than cancer, such as inflammatory bowel disease^9^; and also for people with psychological and physical disabilities.^39^ These results, especially the beneficial effect on improving anxiety and depression, are consistent across the studies, regardless of the participants' characteristics, such as gender, cancer type, or treatment status.

Regarding the QoL, the results are inconsistent. A similar number of studies show beneficial effects (*n* = 2), no significant effect (*n* = 3), and another systematic review show both beneficial and no significant effect of MBSR/MBCT/MBCR. The studies exploring the effect on QoL include both patients and survivors and participants who had already finished their cancer treatment. In the context of cancer diagnosis, treatment, or survivorship, most of these areas might be threatened by the fear of dying, FCR, treatment burden, and it could be that MBSR/MBCT/MBCR are not able to “buffer” these stressors. It might be that this heterogeneity or differences in the percentage of patients versus survivors (not reported in the reviews) might be related to these results. Moreover, the instruments used varied across studies: some are general instruments of QoL, others are specific to cancer. Thus, general instruments might be unable to capture the specific cancer‐related QoL concerns or evaluate physical symptoms that are difficult to change with MBs interventions. Regarding the FCR, the results indicate support for the beneficial effect of MBSR/MBCT/MBCR, thus highlighting the clinical value of mindfulness, even after people have survived the disease. FCR is highly frequent among cancer survivors, being estimated that more than half experience FCR moderate and high intensity.^40^ The intensity of FCR has been linked with dysfunctional levels of worry in all‐day life, affecting well‐being and emotional and social functioning.^41^ The studies included in this meta‐review exploring this outcome were published recently. Other recent studies have conceptualized an MBCT program targeting FCR^42^ and tested its effectiveness,^43^, ^44^ with results remaining 4 weeks after the intervention.^43^ Thus, it could be that mindfulness programs targeting this specific concern would be of great clinical value to cancer survivors.

Results on biological outcomes point in the same direction as psychological outcomes. Although studies explored the biological outcomes have found small effects, mindfulness seems to have a beneficial and significant effect on inflammatory indicators of cancer, as supported by the increase in the immune‐ and inflammatory‐related markers. This is in line with the reported overall results stating that mindfulness might be associated with a positive outcome regarding these biological markers in cancer patients. Nevertheless, the results might be biased by the reduced number of studies addressing these outcomes. As with psychological outcomes, the evidence is not sufficiently solid to support clinical‐relevant effects across time. Even though the evidence indicates that there are beneficial effects on psychological outcomes measured in shorter follow‐ups, the studies on longer follow‐ups showed that the effects tend to disappear. Moreover, the presence of confounders is not controlled in the majority of studies when exploring associations between psychosocial factors and, for example, inflammatory markers, controlling for age, sex, socioeconomic status, alcohol use, sleep quality should be controlled; as well as assessed menopausal, diet, fitness status.^45^ Future studies should follow these recommendations,^45^, ^46^ which might contribute to more solid evidence. Some heterogeneity was observed in the length of follow‐up and results (how these follow‐up times are pooled in meta‐analysis). There seems to be a trend of beneficial short effects of MBSR/MBCT/MBCR, but it seems inconsistent when we analyzed longer follow‐up times (e.g., 12 months). Only studies using RCTs and comparing the long‐term effects on psychological and biological outcomes, as well as long‐term prevalence of cancer relapse would help bring a clearer picture of these mechanisms. Future studies should also evaluate the long‐term effect of continued home practice comparing to those stopping just after the end of the programs. In addition, long‐term studies might help to better understand the effects, especially comparing those survivors with high and low FCR.

### 4.1 Study limitations

Although numerous reviews address the effects of MBSR/MBCT/MBCR, the evidence is based on some overlapping studies, which may bias the results found. For this reason, one review was excluded since all primary studies included were presented in other reviews. Our overlapping analysis showed us that, on average, the same primary study was included twice. Even though the overlapping seems not to be highly significant, results should be interpreted with caution.

Importantly, although only studies that included and reported separated results for cancer patients and survivors were included, some bias might occur with the results found. The inclusion and exclusion criteria were diverse among the studies included in this meta‐review. For example, some included only breast cancer patients, others included other cancer types, and some included both. Although we only retained the studies in the reviews that met all of our criteria, the samples characteristics were slightly different across the studies that examined the effectiveness of MBSR/MBCT/MBCR. Half of the studies focused exclusively on breast cancer, and as a consequence, female patients are overrepresented. This might contribute to biased results: on the one hand, women seem to participate more in research, and an effect of social desirability might happen; on the other hand, due to the hormonal contribution of menopause, the results might be different among those in before and after menopausal phase.^45^ However, the majority of the reviews included found low heterogeneity in the sample characteristics. In general, the studies included estimated a low risk of bias, and sensitivity analysis showed robust short‐term results. The majority reported moderated quality of the primary studies included. However, some also reported the impossibility of running sensitivity and risk of publication bias analysis due to the low number of studies included.^33^ This meta‐review highlighted the need for future studies exploring the differences between different age groups, cancer type, and treatment status as moderators of the effects of MBSR/MBCT/MBCR.

Additionally, the methodological options of the included studies may have introduced some bias, such as the use of different control groups: “white” control groups (waitlist; no intervention) and active control groups may also contribute to the above‐mentioned bias. The treatment and procedures offered as “usual” care might significantly differ between cancer hospitals or even more between different settings (hospital vs. other settings). It should be noted that studies with passive control conditions (compared with active/competing conditions) seemed to report more beneficial results.^31^, ^33^ Further studies might focus on studying the common factors between these interventions. Evidence has shown that common factors such as alliance and empathy are determinant factors for the effectiveness of different psychological interventions.^47^ In this way, future studies exploring the role of these variables might contribute to a more accurate “picture” of these effects.

### 4.2 Implications for clinical practice and future research

This meta‐review provides a broad perspective on the available evidence regarding MBCT/MBSR/MBCR. We reviewed studies written in English, Spanish, French, or Portuguese, which is a strength that should be highlighted. We expect to contribute to future study design. The diverse approaches used among studies, mainly regarding procedures and methodological options, precluded consistent and high‐quality evidence. This meta‐review highlighted the importance of targeting different populations based on their cancer type, age, and education level. A further step on research in this field would be to test the effectiveness of MBSR/MBCT/MBCR on different populations to identify those who would benefit more and how, exploring the moderators. Furthermore, it would be interesting to determine if MBSR/MBCT/MBCR effects are clinically relevant, whether an improvement in depression or anxiety or telomerase activity is clinically significant, affecting health status.

In conclusion, this meta‐review found beneficial effects of MBCT/MBSR/MBCR on psychological and biological outcomes in the short term, but less robust results in long follow‐ups. These results were consistent across patients and survivors, regardless of the cancer type and treatment status. Future studies should focus on developing high‐quality studies with longer follow‐ups, exploring the moderating effects that might contribute to biased results, and on who might benefit more from MBSR/MBCT/MBCR interventions.

## CONFLICT OF INTEREST STATEMENT

The authors reported no conflict of interest.

## AUTHOR CONTRIBUTIONS

Study design (protocol development and registration), literature searches, blind rating of studies to include in the review, data analysis, review of literature, and manuscript redaction and preparation and revision: Juliana Pedro. Interpretation of the data, revision, and approval of the final version of the manuscript: Carina Carvalho‐Maia. Interpretation of the data, revision, and approval of the final version of the manuscript: Sara Monteiro‐Reis. Participation in study design, interpretation of the data, revision, and approval of the final version of the manuscript: Rui Henrique. Participation in study design, interpretation of the data, revision, and approval of the final version of the manuscript: Carmen Jerónimo. Protocol development, blind rating of studies to include in the review, data analysis, and manuscript redaction and preparation, and final approval of the manuscript: Eunice R. Silva. All authors made significant contributions to critically revising the manuscript and approved the final version for submission.

## Supporting information

Supporting information 1

## Data Availability

Data sharing is not applicable to this article as no new data were created or analyzed in this study.
