# Supplementary material for: Evidence of psychological and biological effects of structured Mindfulness‐Based Interventions for cancer patients and survivors: A meta‐review
Source: Psychooncology. 2021 Jul 28;30(11):1836–48. doi: 10.1002/pon.5771 (PMC9290489; doi:10.1002/pon.5771)
Supplement: Supplementary file 1 — Supporting information 1 [file PON-30-1836-s001.docx]

| Supplemental Table 1. Overlap between studies included. | | | | | | | | | | | | |
| --- | --- | --- | --- | --- | --- | --- | --- | --- | --- | --- | --- | --- |
|  | Calero 2018 | Cillensen 2019 | Cramer 2012 | Ford 2020 | Haller 2017 | Piet 2012 | Schell 2019 | Xunlin 2020 | Zhang Jun 2016 | Zhang Mei Fen 2015 | Zhang Qiuxiang 2019 | Number of reviews including the study |
| Andersen et al., 2013 | X |  |  |  | X |  |  |  |  |  |  | 2 |
| Blaes et al., 2016 |  | X |  |  |  |  |  | X |  |  |  | 2 |
| Bower et al., 2014 |  |  |  |  |  |  |  |  | X |  |  | 1 |
| Bower et al. 2015 |  |  |  |  | X |  | X |  |  |  |  | 2 |
| Boyle et al., 2017 |  |  |  |  |  |  |  |  |  |  |  |  |
| Bränström et al. 2010 |  | X |  |  |  | X |  |  |  | X |  | 3 |
| Bränström et al. 2012 |  |  |  |  |  |  |  | X |  |  |  | 1 |
| Bruggeman‐Everts et al. 2017 |  | X |  |  |  |  |  |  |  |  |  | 1 |
| Bisseling et al., 2017 |  |  |  |  |  |  |  |  |  |  | X | 1 |
| Carlson et al., 2002 |  |  |  |  |  |  |  |  |  |  | X | 1 |
| Carlson et al. 2013 (Schellekens et al.2017) |  | X |  |  | X |  |  | X |  |  |  | 3 |
| Carlson et al., 2016 | X |  |  |  | X |  |  | X |  |  |  | 3 |
| Chambers et al. 2017 |  | X |  | X |  |  |  | X |  |  |  | 3 |
| Compen et al. 2018 |  | X |  |  |  |  |  |  |  |  |  | 1 |
| Foley et al. 2010 |  | X |  | X |  | X |  | X |  | X |  | 5 |
| Garland et al. 2014, 2015 |  | X |  | X |  |  |  | X |  |  |  | 3 |
| Hebert 2011 |  |  | X |  |  |  |  |  |  |  |  | 1 |
| Hawkeset al. 2013 |  |  |  |  |  |  |  | X |  |  |  | 1 |
| Hawkeset al. 2014 |  |  |  |  |  |  |  | X |  |  |  | 1 |
| Henderson et al., 2011 |  |  | X |  |  |  |  |  |  |  |  | 1 |
| Henderson et al., 2012 |  | X |  |  | X |  |  | X |  |  |  | 3 |
| Henderson et al., 2013 |  |  |  |  |  |  | X |  | X | X | X | 4 |
| Hoffman et al., 2012 | X | X |  |  | X | X |  | X | X | X | X | 8 |
| Jang et al., 2016 |  | X |  |  |  |  |  | X |  |  |  | 2 |
| Johannsen et al., 2016; Johannsen et al., 2018 |  | X |  |  | X |  |  | X |  |  |  | 3 |
| Johannsen 2015 |  |  |  |  |  |  | X |  |  |  |  | 1 |
| Johns et al.,2012 |  |  |  |  |  | X |  |  |  |  |  | 1 |
| Johns et al.,2014 |  |  |  |  |  |  | X |  |  |  |  | 1 |
| Johns et al.,2015 |  | X |  |  |  |  |  | X |  |  |  | 2 |
| Johns Brown et al., 2016; Johns Von Ah et al., 2016 |  | X |  |  |  |  |  |  |  |  | X | 2 |
| Kenne et al., 2017 |  |  |  |  |  |  | X | X |  |  |  | 2 |
| Kingston et al., 2015 |  | X |  |  |  |  |  | X |  |  |  | 2 |
| Kingston et al., 2012 |  |  |  |  |  | X |  |  |  |  |  | 1 |
| Lee 2017 |  |  |  |  |  |  |  |  |  |  | X | 1 |
| Lehto 2015 |  |  |  | X |  |  |  |  |  |  |  | 1 |
| Lengacher 2009 |  | X | X |  | X | X | X | X | X |  |  | 7 |
| Lengacher 2011  Reich RR, Post–White |  |  | X |  |  |  |  |  |  |  |  | 1 |
| Lengacher , Kip, Post-Whine 2011 |  |  | X |  |  |  |  |  |  |  |  | 1 |
| Lengacher et al., 2012 |  |  |  |  | X |  |  |  |  |  |  | 1 |
| Lengacher, Reich RR 2014 | X |  |  |  |  |  | X |  | X |  |  | 3 |
| Lengacher , Shelton MM, 2014 |  |  |  |  |  |  |  |  |  |  |  |  |
| Lengacher Reich RR, Kip KE,2015 | X |  |  |  |  |  |  |  |  |  |  | 1 |
| Lengacher, Reich RR, Paterson et al., 2015 | X |  |  |  | X |  |  |  |  |  |  | 2 |
| Lengacher et al., 2016 | X | X |  |  | X |  |  | X |  |  | X | 5 |
| Lerman et al., 2011 |  |  |  |  |  | X |  |  |  |  |  | 1 |
| Lerman et al., 2012 |  | X |  |  | X |  | X | X |  |  |  | 4 |
| May et al., 2016 |  |  |  |  |  |  |  | X |  |  |  | 1 |
| Matchim et al., 2010 |  |  |  |  |  |  |  |  |  |  | X | 1 |
| Matchim et al., 2011 |  |  |  |  |  |  |  |  |  |  |  |  |
| Matousek et al., 2010 |  |  |  |  |  |  |  |  |  |  | X | 1 |
| Monti et al., 2006 |  | X |  |  |  |  |  |  |  |  |  |  |
| Monti et al., 2013 |  |  |  |  |  |  |  | X |  |  |  | 1 |
| Nouzouri et al., 2017 |  |  |  |  |  |  |  | X |  |  |  | 1 |
| Nakamura et al., 2013 |  | X |  | X |  |  |  |  |  |  |  | 2 |
| Rahmani et al., 2014 |  |  |  |  |  |  |  |  |  |  | X | 1 |
| Reich et al., 2014 |  |  |  |  |  |  |  |  | X |  |  | 1 |
| Reich et al., 2016 |  |  |  |  |  |  |  |  |  |  | X | 1 |
| Reich et al., 2017 | X | X |  |  |  |  |  |  |  |  |  | 2 |
| Reynolds et al., 2017 |  | X |  |  |  |  |  |  |  |  |  | 1 |
| Schellekens et al.,2017 |  |  |  | X |  |  |  | X |  |  |  | 2 |
| Kenne Sarenmalm et al., 2017 |  | X |  |  |  |  |  |  |  |  |  | 1 |
| Speca et al., 2000 |  | X |  | X |  | X |  |  |  | X |  | 4 |
| Shapiro et al., 2003 |  |  |  |  |  |  | X |  |  |  |  | 1 |
| Victorson et al., 2016 |  |  |  | X |  |  |  |  |  |  |  |  |
| Wang et al., 2017 |  |  |  |  |  |  |  |  |  |  | X | 1 |
| Würtzen et al. 2012 |  |  |  |  |  | X |  |  |  |  |  |  |
| Würtzen et al. 2013;  Würtzen et al. 2015;  Andersen et al. 2013 |  | X |  |  |  | X | X | X | X |  |  | 4 |
| Witek-Januske, 2008 |  |  |  |  |  |  |  |  |  |  |  |  |
| Zernicke et al., 2014 |  | X |  | X |  |  |  | X |  |  |  | 3 |
| Zaidi et al., 2019 |  |  |  |  |  |  | X |  |  |  |  | 1 |
| Zhang et al., 2017 | X | X |  |  |  |  | X | X |  |  |  | 4 |
| Zhang et al., 2016 |  |  |  | X |  |  |  |  |  |  | X | 2 |

Note. When multiple studies are presented in the same line, it indicates that studies use the “same” sample/same trial, and are counted as a single study.
